# A smoothed boundary bidomain model for cardiac simulations in anatomically detailed geometries

**DOI:** 10.1371/journal.pone.0286577

**Published:** 2023-06-09

**Authors:** Niccolò Biasi, Paolo Seghetti, Matteo Mercati, Alessandro Tognetti

**Affiliations:** 1 Information Engineering Department, University of Pisa, Pisa, Italy; 2 Health Science Interdisciplinary Center, Scuola Superiore Sant’Anna, Pisa, Italy; 3 National Research Council, Institute of Clinical Physiology, Pisa, Italy; 4 Research Centre “E. Piaggio”, University of Pisa, Pisa, Italy; University of Southern California, UNITED STATES

## Abstract

This manuscript presents a novel finite difference method to solve cardiac bidomain equations in anatomical models of the heart. The proposed method employs a smoothed boundary approach that represents the boundaries between the heart and the surrounding medium as a spatially diffuse interface of finite thickness. The bidomain boundary conditions are implicitly implemented in the smoothed boundary bidomain equations presented in the manuscript without the need of a structured mesh that explicitly tracks the heart-torso boundaries. We reported some significant examples assessing the method’s accuracy using nontrivial test geometries and demonstrating the applicability of the method to complex anatomically detailed human cardiac geometries. In particular, we showed that our approach could be employed to simulate cardiac defibrillation in a human left ventricle comprising fiber architecture. The main advantage of the proposed method is the possibility of implementing bidomain boundary conditions directly on voxel structures, which makes it attractive for three dimensional, patient specific simulations based on medical images. Moreover, given the ease of implementation, we believe that the proposed method could provide an interesting and feasible alternative to finite element methods, and could find application in future cardiac research guiding electrotherapy with computational models.

## Introduction

Heart diseases remain the leading cause of death worldwide and still cause millions of victims every year. According to the 2022 statistics from the American Heart Association [1], sudden cardiac death appeared on 13.0% of death certificates in 2019 in the USA. Sudden cardiac arrest is thought to be due to ventricular arrhythmias, particularly fibrillation episodes. Cardiac fibrillation is the disruption of the organized cardiac electrical activity into disorganized, self-sustained electrical activation patterns. Cardiac defibrillation proved to be the most effective therapy in preventing sudden cardiac death [2], however the discharge of high electric shocks remains a painful experience associated with severe psychological distress [3, 4]. Therefore, cardiac defibrillation, especially at low energy, remains an interesting research topic. In the last decades, cardiac research recognized computer models as fundamental tools in understanding both cardiac function and its response to external stimulation. Indeed, several recent computational studies focus on the development of carefully designed, low-energy stimulation strategy [5–8].

To date, the bidomain formulation [9] is the gold standard for the representation of cardiac tissue. In the bidomain framework, the heart tissue is represented as the coupling of the intracellular and extracellular spaces through the cell membrane. If the intracellular and extracellular anisotropy ratios are the same, the bidomain model reduces to the much simpler monodomain model [10]. This approximation is often made in large 3D cardiac models to still gain valuable insights while simplifying the problem considerably (see e.g, [11–14]). However, modelling extracardiac stimulation requires that the heart tissue is represented with the more complex, but more accurate, bidomain model [11, 15]. For example, Sepulveda et al. [16] demonstrated that the tissue response in the vicinity of a strong unipolar stimulus results in the simultaneous occurrence of both depolarizing and hyperpolarizing effects, only if the anisotropy ratios between intracellular and extracellular spaces are unequal. This phenomenon, known as virtual electrode polarization, has been well documented in animal experiments [17–19].

Previous works showed that the complex shape of the heart plays an important role in determining the spatiotemporal evolution of the transmembrane potential [20, 21]. Thus, the use of anatomically detailed heart geometries improves the reliability of simulation results. Moreover, patient-specific computational cardiac models have the potentiality to dramatically improve diagnosis and treatment of cardiac pathologies [22]. Recent studies employed high-resolution magnetic resonance imaging not only to characterize cardiac morphology to use in computational models but also to identify altered cardiac functions [14, 23, 24]. The use of anatomical models of the heart requires the accurate solution of bidomain boundary conditions on the voltage along complex boundary shapes dividing the heart tissue and the surrounding medium. This issue is not easily addressable in finite difference (FD) algorithms, especially to model the effect of currents applied in the extracellular space. Jagged boundaries, which inevitably form along complex surfaces when structured grids are employed, cause spurious polarization upon delivery of a defibrillation-strength shock [15]. Although some attempts to solve bidomain equations in human anatomical geometries with FD algorithms were made [25, 26], jagged boundaries have severely limited the applicability of FD methods for solving bidomain equations. Bidomain equations are commonly solved by means of finite element [20, 27, 28] or finite volume [14, 29, 30] methods that employ unstructured grids. Indeed, the use of unstructured grids allows smooth representation of complex surfaces. However, the construction of high-quality finite element meshes is a complex task [15, 23].

The aim of this work is to introduce a new FD approach that accurately implements the bidomain boundary conditions in arbitrary geometries. The proposed method is a generalization of the phase field approach that has already been successfully employed in monodomain models [31–33] and a wide variety of other problems, such as solidification dynamics [34], crack propagation [35], and vesicle dynamics [36]. Smoothed boundary methods (SBMs) have the important advantage to circumvents the need of a structured mesh that explicitly tracks the interfaces. Indeed, SBMs introduce an auxiliary field (i.e., the phase field) that spatially distributes the zero-thickness boundaries into a finite thickness diffuse interface. Therefore, boundary conditions are automatically distributed among the grid points residing within the regions of interface in which the phase field varies smoothly. The major contribution of this work is to provide a pipeline for the application of the SBM to the bidomain problem for the first time. We believe that the method proposed here could be especially useful for three-dimensional, patient-specific simulations based on medical images because of its efficiency and flexibility in handling voxel structures. Indeed, the method can be directly applied on segmented, eventually resized, images without performing additional time-consuming operations associated with the construction of structural meshes.

## Materials and methods

### Governing equations

**Fig 1:**
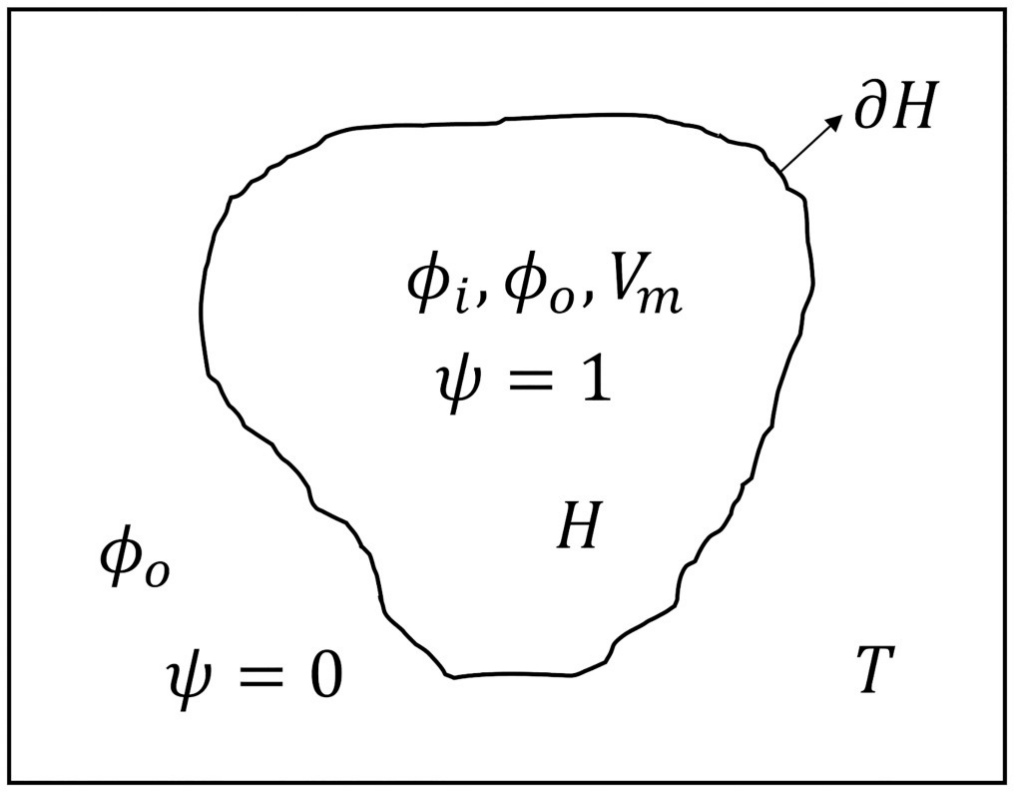
Schematic diagram of the considered geometry. *ϕ*_*i*_ and *V*_*m*_ are defined in the heart domain *H*, whereas *ϕ*_*o*_ is defined both in *H* and in the external conductor *T*. ∂*H* indicates the boundary between the heart and torso domains. The phase field *ψ* introduces a smooth interface between the heart domain (where *ψ* = 1) and the external conductor (where *ψ* = 0).

The bidomain model provides a strategy for understanding larger-scale attributes of the cardiac structure, with its huge number of individual cells, without having to describe that structure in cellular detail. The bidomain model was first proposed and qualitatively described by Schmitt in 1969 [37]. Subsequently, Miller and Geselowitz [38], and Tung [39] proposed a rigorous mathematical formulation of the isotropic bidomain model. Cardiac muscle cells are cylindrical, tend to be arranged in parallel arrays in local regions, and are electrically interconnected in a complex fashion through the gap junctions. These connections between cells exhibit a low resistance in the normal state. Therefore despite its cellular nature, the heart muscle electrically acts in many respects as a syncytium. Thus, the intracellular space can be seen as a continuous domain, or syncytium, with a resistivity which is an appropriate average of the contribution of the cell interior (i.e., the cytoplasm) and that of the gap junctions. A second domain is the fluid matrix which forms a continuous extracellular, or interstitial, outer space. In the bidomain model, the heart tissue is represented by the coupling of the intracellular space and the extracellular space. Both intracellular and extracellular spaces take up the entire heart volume *H* (Fig 1). Consequently, the intracellular potential (*ϕ*_*i*_), and extracellular potential (*ϕ*_*o*_) are defined in each point of the cardiac domain *H* and the membrane potential can be defined as:

$$\begin{array}{c} V_{m}=\phi_{i}-\phi_{o} \end{array}$$ (1)

Moreover, *ϕ*_*i*_ and *ϕ*_*o*_ are spatially averaged continuous electric potential fields, thus their behaviour can be described by the following partial differential equations coupled with an appropriate set of boundary conditions [40, 41]:

$$\begin{array}{c} \frac{\partial V_{m}}{\partial t}-\nabla \cdot (D_{i}\nabla (V_{m}+\phi_{o}))=-I_{ion}(V_{m},w) \end{array}$$ (2)

$$\begin{array}{c} \nabla \cdot ((D_{i}+D_{o})\nabla \phi_{o})+\nabla \cdot (D_{i}\nabla V_{m})+I_{ext}=0 \end{array}$$ (3)

$$\begin{array}{c} \frac{\partial w}{\partial t}=f(V_{m},w) \end{array}$$ (4)

where *D*_*i*_, *D*_*o*_ are the diffusivity tensors of the intracellular and extracellular space. *I*_*ext*_ is the external applied current source, whereas *I*_*ion*_ represents the ionic transmembrane current. The ionic term is related to the state of the cellular membrane, described by a set of variables *w*, through an ionic model. In this work, we employed our previously published phenomenological model of cardiac cells [42] to describe the ionic current. The components of the diffusivity tensor are determined by the tissue conductivities and local orientation of cardiac fibers. If the fiber direction is given by the vector *f*, a diffusion tensor can be written as:

$$\begin{array}{c} D=D_{⊥}I+(D_{\|}-D_{⊥})ff^{T} \end{array}$$ (5)

where *D*_⊥_ and *D*_∥_ are the diffusivity values in the transverse and longitudinal directions of the fibers, respectively. The definition of Eq (5) applies for both *D*_*i*_ and *D*_*o*_. In this work, the longitudinal and transverse diffusivity values for intracellular and extracellular space are set to: *D*_∥*i*_ = 2.4 *cm*^2^/*s*, *D*_∥*o*_ = 2.4 *cm*^2^/*s*, *D*_⊥*i*_ = 0.35 *cm*^2^/*s*, *D*_⊥*o*_ = 2 *cm*^2^/*s*. They are obtained from the conductivity values reported in [43] considering a membrane capacitance of 1 *uF*/*cm*^2^ and a surface to volume ratio equal to 1000 *cm*^−1^.

In the general case, the cardiac tissue is surrounded by a passive external conductor *T* (i.e., the torso). Typical boundary conditions between heart and torso (i.e., on ∂*H* in Fig 1) impose electrical continuity between the external conductor and the extracellular medium, and electrical insulation between the external conductor and the intracellular medium [40, 41, 44]:

$$\begin{array}{c} n_{h}\cdot D_{o}\nabla \phi_{o}=n_{h}\cdot D_{t}\nabla \phi_{t} \end{array}$$ (6)

$$\begin{array}{c} \phi_{t}=\phi_{o} \end{array}$$ (7)

$$\begin{array}{c} n_{h}\cdot D_{i}\nabla V_{m}=-n_{h}\cdot D_{i}\nabla \phi_{o} \end{array}$$ (8)

where *D*_*t*_ is the diffusivity tensor in the torso. In all the simulations, we considered the external conductor isotropic with *D*_*t*_ = 4 *cm*^2^/*s*. Note that the external electric potential *ϕ*_*t*_ can be treated as an extension of the extracellular potential *ϕ*_*o*_ [41]. Thus, the governing equation in the passive external conductor can be expressed as:

$$\begin{array}{c} \nabla \cdot (D_{t}\nabla \phi_{o})=I_{ext} \end{array}$$ (9)

where *ϕ*_*o*_ is now defined in the whole space indicating the extracellular potential in the heart, and the external potential in the torso. An additional boundary condition imposing zero current flux outside the torso geometry is also required:

$$\begin{array}{c} n_{t}\cdot D_{t}\nabla \phi_{o}=0 \end{array}$$ (10)

Eqs (1)–(9) provide a complete formulation of the bidomain model.

### The phase field approach

For simplified geometries, such as two-dimensional sheets and three dimensional slabs of tissue, the implementation of the boundary conditions (6), (7), (8), (10) is straightforward, even when including tissue anisotropy (e.g., [45, 46]). However, when considering more complicated geometries with curved boundaries and complex fiber orientations, the aforementioned boundary conditions are not easy to implement in a FD scheme. In this work, we employed the SBM [31, 47] to implicitly implement bidomain boundary conditions in irregular geometries. In SBMs, the internal domain boundaries are described by an auxiliary field *ψ* which takes a value of 1 inside the domain of interest and 0 outside. Therefore, the sharp domain boundary is smoothed to yield a finite thickness interface, where *ψ* varies smoothly between 0 and 1. Additionally, ∇*ψ*/|∇*ψ*| provides an approximation of the inward normal vector in the zero-thickness limit. Notably, the precise shape of the phase field profile in the thin interface region is not critical for SBMs, as shown in previous works [31, 32, 47, 48]. We generated *ψ* by integrating an auxiliary diffusion equation until a steady state is achieved [31, 32]:

$$\begin{array}{c} \frac{\partial \psi }{\partial t}=\xi^{2}\nabla^{2}\psi +\frac{\left(2\psi -1\right)}{2}-\frac{{\left(2\psi -1\right)}^{3}}{2} \end{array}$$ (11)

with initial conditions *ψ* = 1 in the heart domain, and *ψ* = 0 outside (Fig 1). The parameter *ξ* controls the width of the diffusive interface, which is approximately 4*ξ* [31]. In this work, we used a value of *ξ* equal to 0.025 cm, if not otherwise stated. The auxiliary diffusion Eq (11) was integrated with a second order central FD scheme and the forward Euler method. Considering a generic partial differential equation, the phase field approach consists of extending the problem to the entire computational domain by multiplying each term by the phase field. Then, by using the product rule and by exploiting ∇*ψ*/|∇*ψ*| as an approximation of the inward normal vector, the equation can be rewritten in terms of the imposed flux at the domain boundary (see [47] for further details). The partial differential equation obtained is defined in the entire computational domain and implicitly implements Neumann boundary conditions. A similar procedure also allows to implement Dirichlet or mixed boundary conditions [47].

### Smoothed boundary bidomain model

In this work, we applied the phase field approach to the bidomain equations to obtain a Smoothed Boundary Bidomain (SBB) formulation. Following the approach described in [47], the Eq (2) can be extended to the entire computational domain by multiplying all the terms of the equation by *ψ*. Then, by applying the product rule, the following expression is obtained:

$$\begin{array}{c} \psi \frac{\partial V_{m}}{\partial t}-\nabla \cdot (\psi D_{i}\nabla (V_{m}+\phi_{o}))+\nabla \psi \cdot (D_{i}\nabla (V_{m}+\phi_{o}))=-\psi I_{ion}(V_{m},w) \end{array}$$ (12)

Because the inward unit normal of the boundary is given by ∇*ψ*/|∇*ψ*|, the boundary condition (8) implies ∇*ψ* ⋅ (*D*_*i*_∇ (*V*_*m*_ + *ϕ*_*o*_)) = 0. Thus, we obtain:

$$\begin{array}{c} \psi \frac{\partial V_{m}}{\partial t}-\nabla \cdot (\psi D_{i}\nabla (V_{m}+\phi_{o}))=-\psi I_{ion}(V_{m},w) \end{array}$$ (13)

This equation implicitly implements the boundary condition (8) (see S1 Appendix for a simple proof in the 1D case). Notably, Eq (13) does not have to be solved for the entire computational domain, but only for nodes associated to values of *ψ* greater than a threshold (i.e., internal nodes). As in [31], we fixed this threshold to 10^−4^. Similarly, we can extend Eq (3) to the whole computational domain by multiplying each term by *ψ*. Instead, Eq (9) is defined only outside the heart domain, thus it should be multiplied by (1 − *ψ*). At this point, the two equations are summed to provide a single equation instantaneously relating *V*_*m*_ and *ϕ*_*o*_ in the whole computational domain:

$$\begin{array}{c} \psi \nabla \cdot (D_{i}\nabla V_{m})+\psi \nabla \cdot ((D_{i}+D_{o})\nabla \phi_{o})+(1-\psi )\nabla \cdot (D_{t}\nabla \phi_{o})=I_{ext} \end{array}$$ (14)

Again, by using the product rule and the fact that the inward unit normal of the boundary is given by ∇*ψ*/|∇*ψ*| the equation can be rewritten as:

$$\begin{array}{c} \nabla \cdot (\psi D_{i}\nabla V_{m})+\nabla \cdot ((\psi (D_{i}+D_{o})+(1-\psi )D_{t})\nabla \phi_{o})=I_{ext} \end{array}$$ (15)

which implicitly satisfies the sum of boundary conditions (6) and (8) (see S1 Appendix. for a proof in 1D case). Thus, Eqs (13) and (15) allow for the computation of *V*_*m*_ and *ϕ*_*o*_ automatically implementing bidomain boundary conditions (6), (7), (8) between the heart and the torso. As shown in S1 Appendix, and similarly to previous works [31, 47], the principal source of error in the boundary conditions is related to the spatial integral of $\psi (\frac{\partial V_{m}}{\partial t}+I_{ion}(V_{m},w))$ across the interfacial region. To reduce as much as possible the error, we modified Eq (13) in such a way that in the interfacial region the time derivative and ionic terms are not considered, partially recovering a sharp interface:

$$\begin{array}{c} \Theta (\psi -\psi_{thr})\frac{\partial V_{m}}{\partial t}-\nabla \cdot (\psi D_{i}\nabla (V_{m}+\phi_{o}))=-\Theta (\psi -\psi_{thr})I_{ion}(V_{m},w) \end{array}$$ (16)

where Θ is the Heaviside step function and *ψ*_*thr*_ = 0.99 is a threshold on the value of *ψ*. Basically, if *ψ* < *ψ*_*thr*_ then we are in the interfacial region and the equation reduces to ∇ ⋅ (*ψD*_*i*_∇ (*V*_*m*_ + *ϕ*_*o*_)) = 0, which allows for instantaneous and accurate implementation of bidomain boundary conditions. The major drawback is that the equation is not always parabolic, thus its solution requires solving a linear system also when using splitting techniques and explicit integration methods. Notably, such a modification could also be applied on the smoothed boundary monodomain formulation. Nevertheless, it could not be convenient because, as shown in [31], the small error introduced in no flux boundary conditions is not critical for monodomain simulations, whereas it is for bidomain simulations, especially when strong external stimuli are present.

Note that Eq (15) implicitly implements bidomain boundary conditions at heart-torso boundaries ∂*H*, but does not consider arbitrary shaped external conductors. Nevertheless, with a procedure similar to those described above, Eq (15) can be easily generalized to account also for no-flux boundary conditions at the torso external boundaries:

$$\begin{array}{c} \psi_{t}\nabla \cdot (\psi D_{i}\nabla V_{m})+\nabla \cdot (\psi_{t}(\psi (D_{i}+D_{o})+(1-\psi )D_{t})\nabla \phi_{o})=\psi_{t}I_{ext} \end{array}$$ (17)

where *ψ*_*t*_ is an additional phase field that defines the torso domain inside a larger computational box. Again, (17) does not need to be solved in the whole computational domain but only for values of *ψ*_*t*_ higher than a threshold. As for *ψ*, we fixed the threshold for *ψ*_*t*_ to 10^−4^.

### Numerical integration schemes

All the numerical implementation is carried out in MATLAB and runs on a single AMD Ryzen Threadripper 3960X. The SBB Eqs (15) and (16) can be discretized in space and time with any numerical scheme while implicitly implementing the bidomain boundary conditions between the heart and the torso. In this work, we adopted the second-order central FD method with uniform spacing *dx* = 0.025 *cm* for spatial discretization and a semi-implicit time integration scheme [44, 49] with *dt* = 0.02 *ms* (if not otherwise stated). Thus, for a generic timestep *k*, we obtained the following linear system:

$$\begin{array}{c} \left(\Theta_{\psi }-dtA_{s})V_{m}^{k+1}-dtA_{so}\phi_{o}^{k+1}=\Theta_{\psi }V_{m}^{k}-dt\Theta_{\psi }I_{ion}(V_{m}^{k},w^{k}\right) \end{array}$$ (18)

$$\begin{array}{c} A_{sv}V_{m}^{k+1}+B_{s}\phi_{o}^{k+1}=-I_{ext} \end{array}$$ (19)

***A*_*s*_** is the ∇ ⋅ (*ψD*_*i*_∇) operator, and it is a *N*_*in*_ × *N*_*in*_ matrix, where *N*_*in*_ is the number of internal nodes (i.e., where *ψ* > 10^−4^). ***A*_*so*_** is a *N*_*in*_ × *N*_*tot*_ matrix, where *N*_*tot*_ is the total number of nodes, and it is obtained by replicating the columns of ***A*_*s*_** in the column indices corresponding to internal nodes. Similarly, ***A*_*sv*_** is a *N*_*tot*_ × *N*_*in*_ matrix, which is built by replicating the rows of ***A*_*s*_** in the row indices corresponding to internal nodes. **Θ_*ψ*_** is a diagonal matrix, whose diagonal elements corresponds to the element values of Θ(*ψ* − *ψ*_*thr*_). ***B*_*s*_** is a *N*_*tot*_ × *N*_*tot*_ matrix implementing the ∇ ⋅ ((*ψ*(*D*_*i*_ + *D*_*o*_) + (1 − *ψ*)*D*_*t*_)∇) operator. The matrices construction is based on standard second-order central FD scheme [50]. Further details about matrices construction are provided in S1 Appendix. The state variables of the ionic model were updated with the forward Euler method. The coupling of Eqs (18) and (19) provides a system of linear equations which allows to update *V*_*m*_ and *ϕ*_*o*_ at each time step. *V*_*m*_ and *ϕ*_*o*_ can be computed by solving the system in its full form or dividing it into two subproblems by exploiting the operator splitting technique. In this work, we solved the full system for 1D and 2D simulations, whereas we exploited the operator splitting technique for 3D simulations. The operator splitting approach we employed consists of the following 3 steps [41]:

1. Compute $V_{m}^{k+1/2}$ by solving the following system:
  $$\begin{array}{c} (\Theta_{\psi }-dtA_{s})V_{m}^{k+1/2}=\Theta_{\psi }V_{m}^{k}+dtA_{so}\phi_{o}^{k} \end{array}$$ (20)
2. Compute $V_{m}^{k+1}$
  $$\begin{array}{c} V_{m}^{k+1}=V_{m}^{k+1/2}-dt\Theta_{\psi }I_{ion}(V_{m}^{k+1/2},w^{k}) \end{array}$$ (21)
3. Compute $\phi_{o}^{k+1}$ by solving the following system:
  $$\begin{array}{c} B_{s}\phi_{o}^{k+1}=-A_{sv}V_{m}^{k+1}-I_{ext} \end{array}$$ (22)

In both the approaches, the most demanding operation is the solution of the large linear systems {(18), (19)}, (20), and (22). When the systems are relatively small (e.g., for 1D and small 2D models), they can be easily solved with standard routines (e.g., the *mldivide* function of MATLAB). However, when the systems are large preconditioned iterative methods must be used. Therefore, we used a biconjugate gradient stabilized solver with ILU preconditioning (*bicgstab* MATLAB function). To accelerate convergence, the solver uses initial guesses for $V_{m}^{k+1}$ and $\phi_{o}^{k+1}$. In the case of the full coupled system, $V_{m}^{k}$ and $\phi_{o}^{k}$ were used as initial guesses for $V_{m}^{k+1}$ and $\phi_{o}^{k+1}$, respectively. When using the operator splitting, the initial guess for $\phi_{o}^{k+1}$ was $\phi_{o}^{k}+(V_{m}^{k+1}-V_{m}^{k})\delta$, where Δ = *D*_⊥*i*_/(*D*_⊥*i*_ + *D*_⊥*o*_) [25]. Iteration of the solver for the full system was terminated when the relative norm of the residual became smaller than 10^−5^. Iteration of the solver for the system (20) was terminated when the relative norm of the residual became smaller than 10^−6^. Notably, the solver takes only a handful of iterations to converge. Finally, iteration of the solver for the system (22) was terminated when either the relative norm of the residual became smaller than 10^−4^ or the norm of the residual became smaller than 10^−4^((*dx*/0.02 *cm*)^3^*N*_*tot*_)^1/2^, as in [25]. It is worth mentioning that, beyond the standard and well established ILU preconditioner used in this study, several other more efficient choices are possible. In particular, multigrid preconditioners demonstrated to be very powerful and well-suited for large bidomain problems, especially when executed in parallel clusters [26, 41, 51, 52].

We tested the SBB method in three sample geometries. First, we considered a 1D cable 6 cm long with the heart domain placed in the middle (4 cm long). We compared the results obtained with the SBB method with those obtained by solving standard bidomain equations. Second, we employed the smoothed boundary approach in a 2D annular geometry (both isotropic and anisotropic) and we compared the results with those obtained with a standard FD scheme. Indeed, bidomain equations in an annular geometry can be solved easily using finite differences after transforming the equations to polar coordinates. Finally, we tested the SBB method in an anatomically detailed geometry. We considered a left human ventricle with fiber anisotropy [53] immersed in a conductive bath. The cardiac tissue was stimulated through a couple of electrodes placed at the opposite boundaries of the bath. Note that the fiber orientation is only defined inside the heart domain, whereas solving the SBB equations requires fiber orientation also in the interfacial region. Thus, we extrapolated the orientation of the fibers in the interfacial region with a nearest neighbour approach [31].

## Results

### 1D cable model

**Fig 2:**
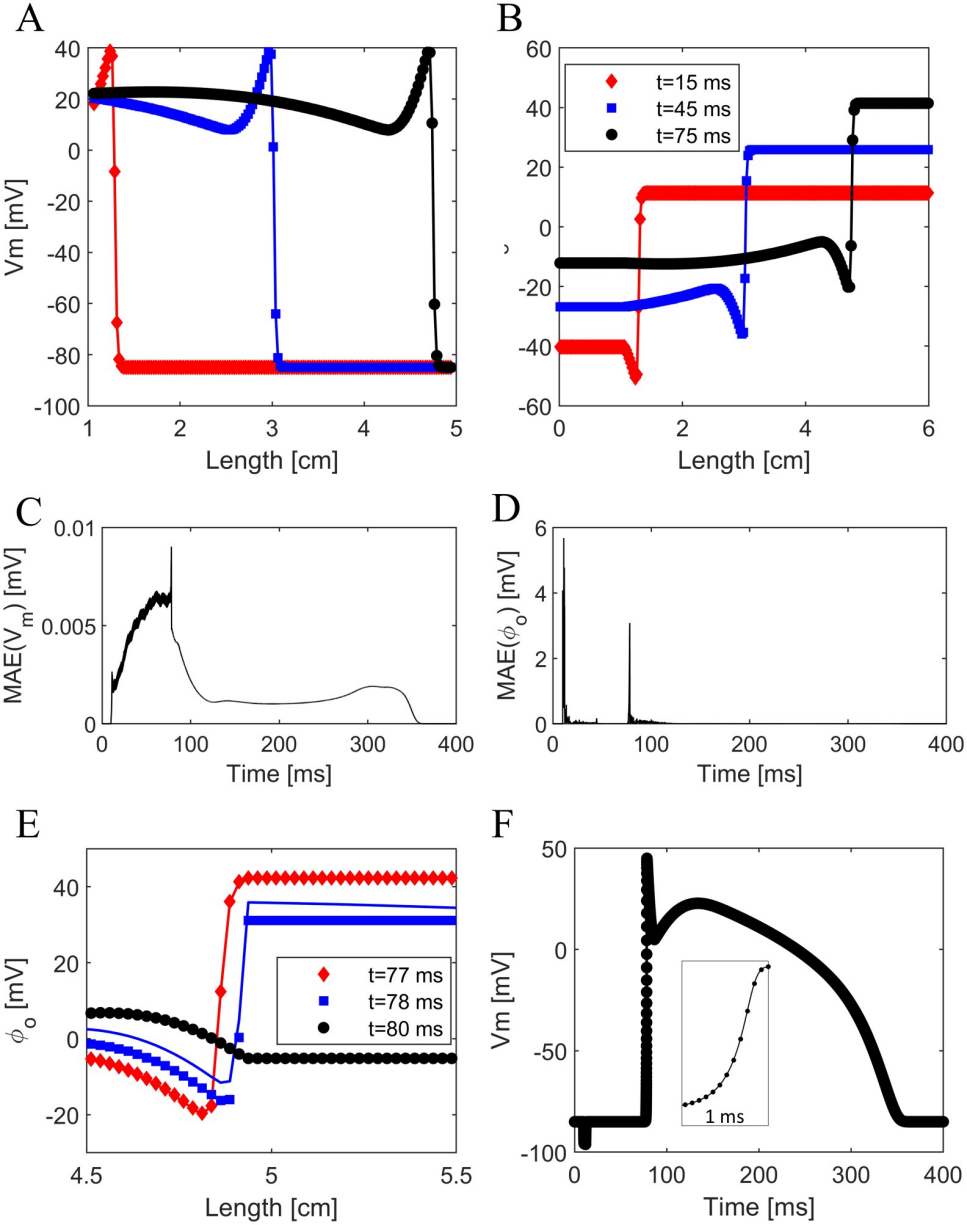
Comparison between SBB solution (colored lines) and the reference solution (filled markers) in a 1D cable model. A) Membrane potential along the cable in three distinct time instants. B) Extracellular potential along the cable in three distinct time instants. C) Mean absolute error in the membrane potential at each time step. D) Mean absolute error in the extracellular potential at each time step. E) Extracellular potential along the cable in three different time instants corresponding to the arrival of the action potential at the right end of the cable. F) Membrane potential in the right end of the cable. The inset zooms on the upstroke phase.

We first applied the SBB method on a simple cable geometry 6 cm long. The heart domain was placed in the center of the cable and was 4 cm long. We stimulated the cable with a defibrillatory shock delivered between the two ends of the cable. The cathode was placed on the left edge of the cable, whereas the anode was placed on the right edge. The stimulation starts at *t* = 10 *ms* and terminates after 2 ms. The whole simulation lasts for 400 ms. Fig 2A and 2B show the resulting cardiac action potential and the corresponding extracellular potential, which propagate along the cable. For comparison, we also report the results obtained by solving bidomain equations with the standard FD method (filled markers in Fig 2). We observed almost complete agreement between the SBB solution and the reference solution. The mean absolute error (MAE) in the computation of *V*_*m*_ is always lower than 0.01 *mV*, as shown in Fig 2C. The maximum error occurs when the action potential reaches the right end of the cable. Indeed, the largest errors in phase field methods are observed when the derivative of the membrane potential and the ionic current are maximum in the interfacial region [31]. The MAE in the extracellular potential can achieve up to 6 mV during the defibrillatory shock (Fig 2D), when *ϕ*_*o*_ varies between about ±3000 *mV*. The MAE also increased significantly when the action potential achieves the right end of the cable. However, excluded these two short time intervals, the MAE in *ϕ*_*o*_ is always lower than 0.6 *mV*. Indeed, as shown in Fig 2E, the extracellular potential deviates from the reference solution only for a short time interval of about 1 *ms* (i.e., the duration of the upstroke). Notably, this discrepancy does not affect the rest of the simulation, as long as it is quickly recovered. Additionaly, the membrane potential does not deviate significantly from the reference solution, even when considering the right end of the cable, where the errors are largest (Fig 2F). Additionally, in the right end of the cable, the relative error in the action potential duration is lower than 10^−4^, whereas the relative error in the maximum upstroke velocity is lower than 10^−3^.

**Fig 3:**
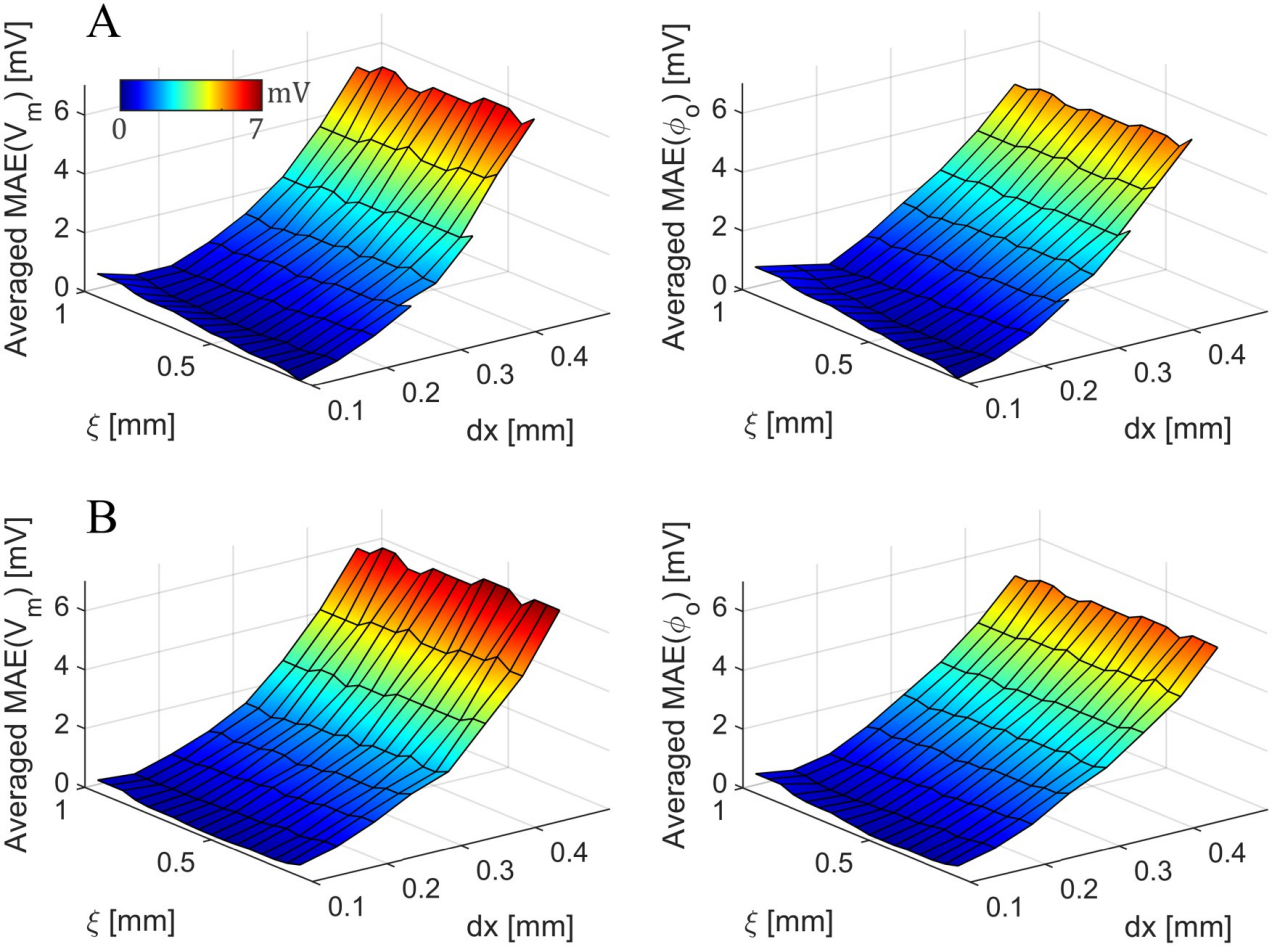
Grid convergence tests of the SBB method for different values of *ξ* with and without operator splitting. A) Time-averaged mean absolute error in membrane potential and extracellular potential obtained with the SBB method without operator splitting for different values of *ξ* and *dx*. Missing elements indicate that the SBB algorithm without operator splitting did not achieve a solution. B) Time-averaged mean absolute error in membrane potential and extracellular potential obtained with the SBB method exploiting operator splitting for different values of *ξ* and *dx*.

To assess the influence of the spatial discretization and interfacial width on the SBB method, we carried out a grid convergence test for different values of *ξ* (Fig 3A). In particular, we simulated the cable model with the SBB method for *dx* varying from 0.01 to 0.045, and *ξ* from 0.015 to 0.1, both in steps of 0.005. For each pair (*dx*, *ξ*), we compared the SBB solution with a reference solution obtained by solving bidomain equations with the standard FD method (*dx* = 0.01 *cm*). We evaluated the discrepancy between two simulations by averaging the MAE in time for both *V*_*m*_ and *ϕ*_*o*_. Since the SBB grid and the reference grid have different resolutions, we interpolated the SBB solution on the reference grid to compute the MAE at each time instant. The grid convergence test showed that the SBB method converges to the reference solution when *dx* and *ξ* approach to 0. Whereas the effect of the spatial discretization is predictable, the role of the interfacial thickness is worth to be investigated. First, when *ξ* is small with respect to the *dx* the algorithm do not converge (missing elements in Fig 3A). This is an expected behaviour already reported in [31]. Similarly, when the value of *ξ* is large the stimulation of the tissue is delayed, and thus the MAE increases in both *V*_*m*_ and *ϕ*_*o*_. To ensure that the operator splitting approach is not affecting the convergence of the algorithm, we performed the same analysis by using the SBB method exploiting operator splitting (Fig 3B). As expected, the discrepancy with the reference solution is slightly higher in this case, but the SBB algorithm is still converging. Surprisingly, for large *ξ* and small *dx* the MAE is lower for the operator splitting approach. This unexpected behaviour is explained as a compensation of errors due to the higher conduction velocity observed with the operator splitting approach that compensate the error due to the delayed stimulation of the tissue. Additionally, for all the tested value of *ξ* and *dx* the SBB method with operator splitting can always achieve a solution. It is worth mentioning that averaged MAE is a good indicator to test the convergence of the algorithm but it is not sufficient to establish the best choice for the value of *ξ*, a procedure that should consider also other error estimates.

### 2D annular model

**Fig 4:**
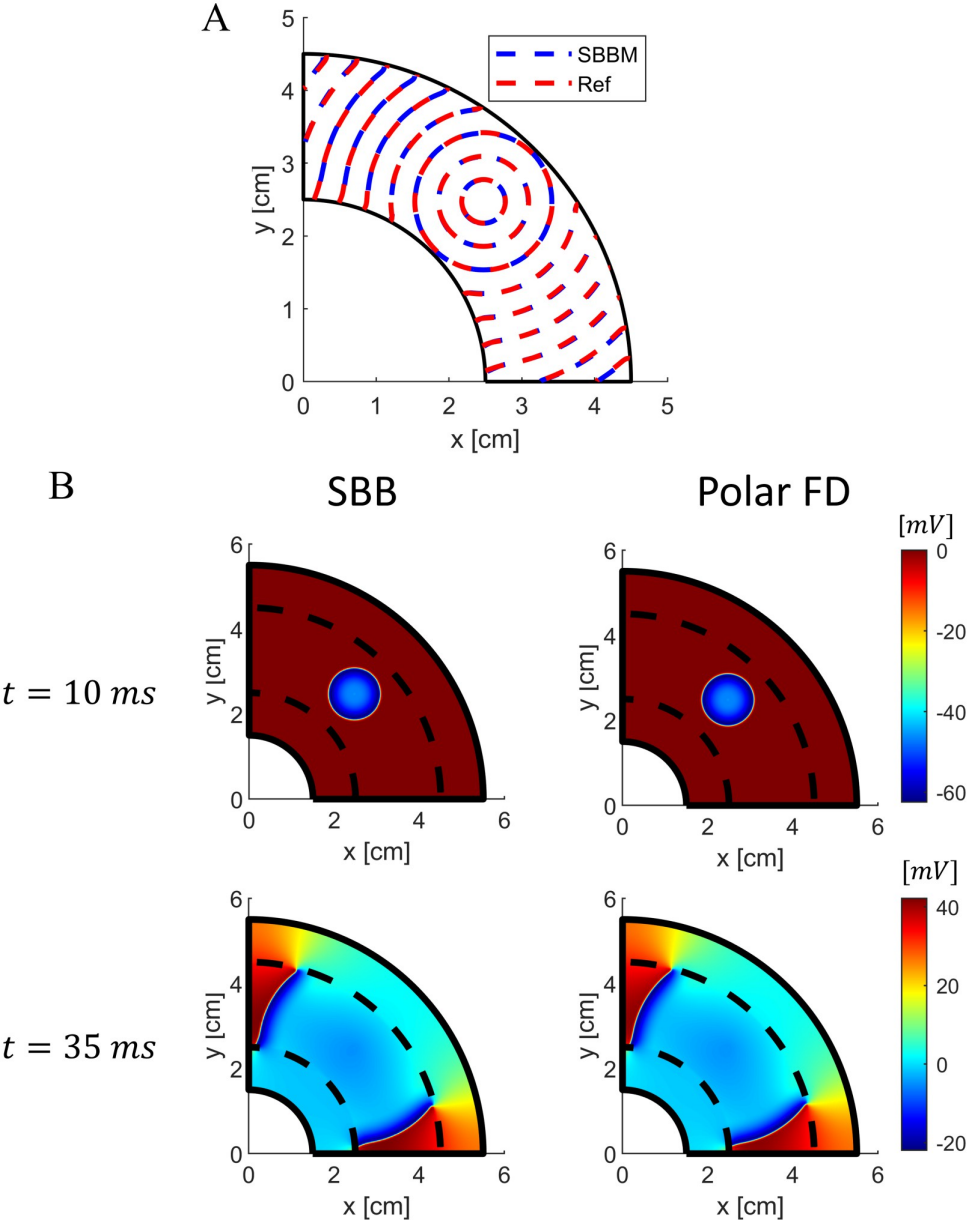
Comparison between SBB and polar FD methods in a 2D isotropic annular geometry with cathodic extracellular stimulation. A) Contour plots of membrane potential (*V*_*m*_ = −40 *mV*) at different times for the SBB and polar FD methods. B) Comparison of extracellular potential field obtained with SBB and polar FD methods at two different time instants. Dashed black lines indicate the boundary between the cardiac tissue and the external conductor.

We first tested the SBB method in a 2D isotropic annular geometry. The tissue was stimulated with an extracellular unipolar cathodic point source (i.e., *I*_*ext*_ < 0). We compared the results with those obtained with a standard FD scheme in polar coordinates. Note that, the polar coordinate discretization leads to a nonuniform grid spacing relative to the cartesian grid used for the SBB method. Thus, to minimize the effect of the different discretization and quantify the discrepancy only between the two approaches, we used smaller grid spacings (i.e., *dx* = 0.1 *mm*, *ξ* = 0.2 *mm*, for the Cartesian grid; *dr* = 0.1 *mm*, *dθ* = 0.0029, for the polar grid). Fig 4A compares the contour *V*_*m*_ = −40 *mV* of the wavefront at different times for the SBB and the polar FD methods. We observed almost complete agreement between wavefront velocity in the two methods. The relative difference between the wavefront velocity obtained with the two methods is everywhere smaller than 1%. Similarly, excellent agreement was observed in the extracellular potential field, as shown in Fig 4B.

**Fig 5:**
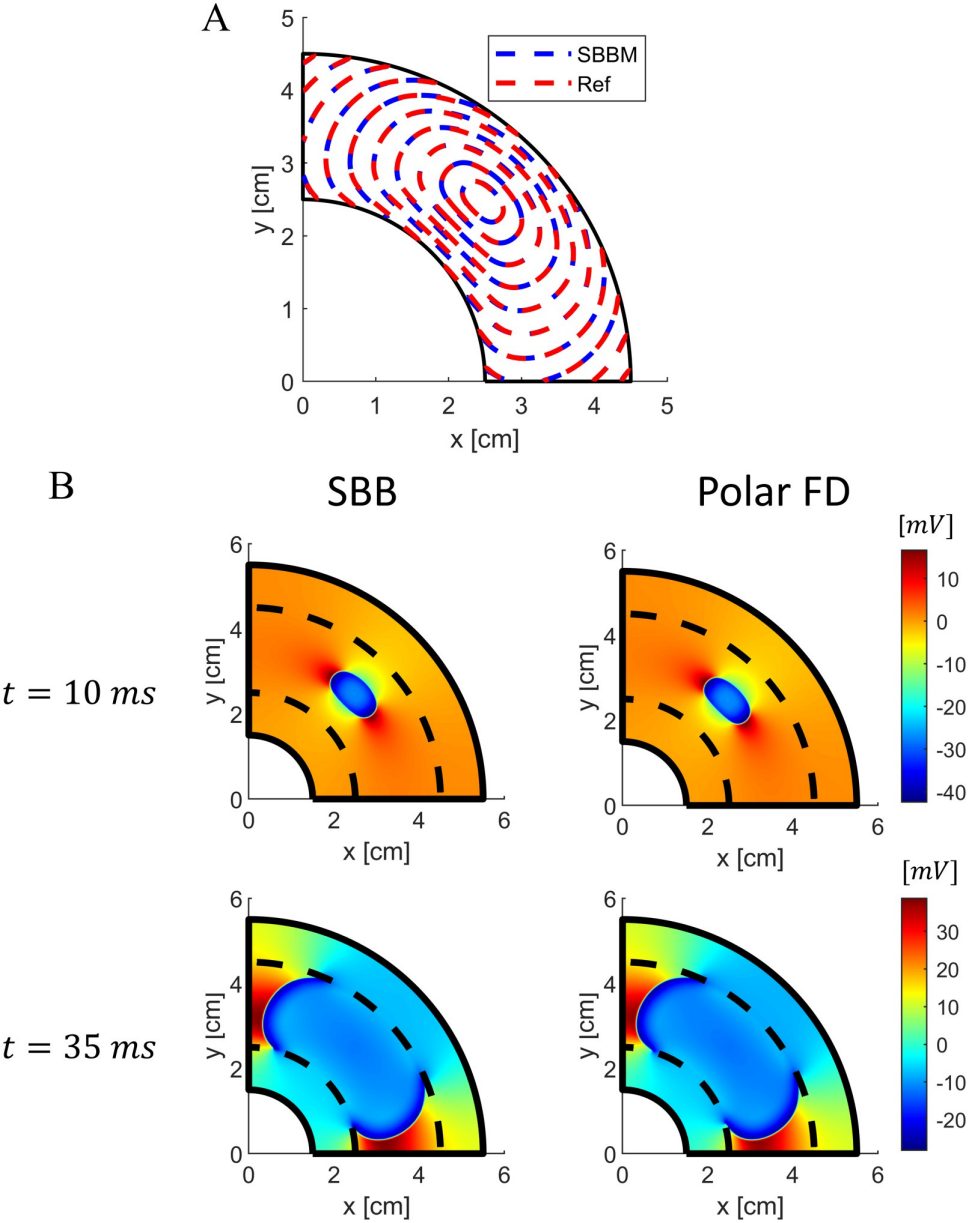
Comparison between SBB and polar FD methods in a 2D anisotropic annular geometry with cathodic extracellular stimulation. A) Contour plots of membrane potential (*V*_*m*_ = −40 *mV*) at different times for the SBB and polar FD methods. B) Comparison of extracellular potential field obtained with SBB and polar FD methods at two different time instants. Dashed black lines indicate the boundary between the cardiac tissue and the external conductor.

Since the cardiac tissue is highly anisotropic, it is important to test the accuracy of the SBB formulation also in anisotropic domains. We considered a 2D annular model with fibers oriented tangentially, which can be simulated easily with the standard FD method in polar coordinates. Again, we observed no significant discrepancy between the proposed SBB formulation and the polar FD method, when the tissue was stimulated with an extracellular cathodic point source (Fig 5). Wavefront velocity is perfectly matched in the fiber direction (Fig 5A). However, in the direction orthogonal to the fibers SBB method results in a slightly larger conduction velocity. The discrepancy is mainly due to different grids (i.e., polar and cartesian) used in the two simulations, and is more evident when the conduction velocity is low. Nevertheless, the relative difference between the wavefront velocity obtained with the two methods is everywhere smaller than 5%.

**Fig 6:**
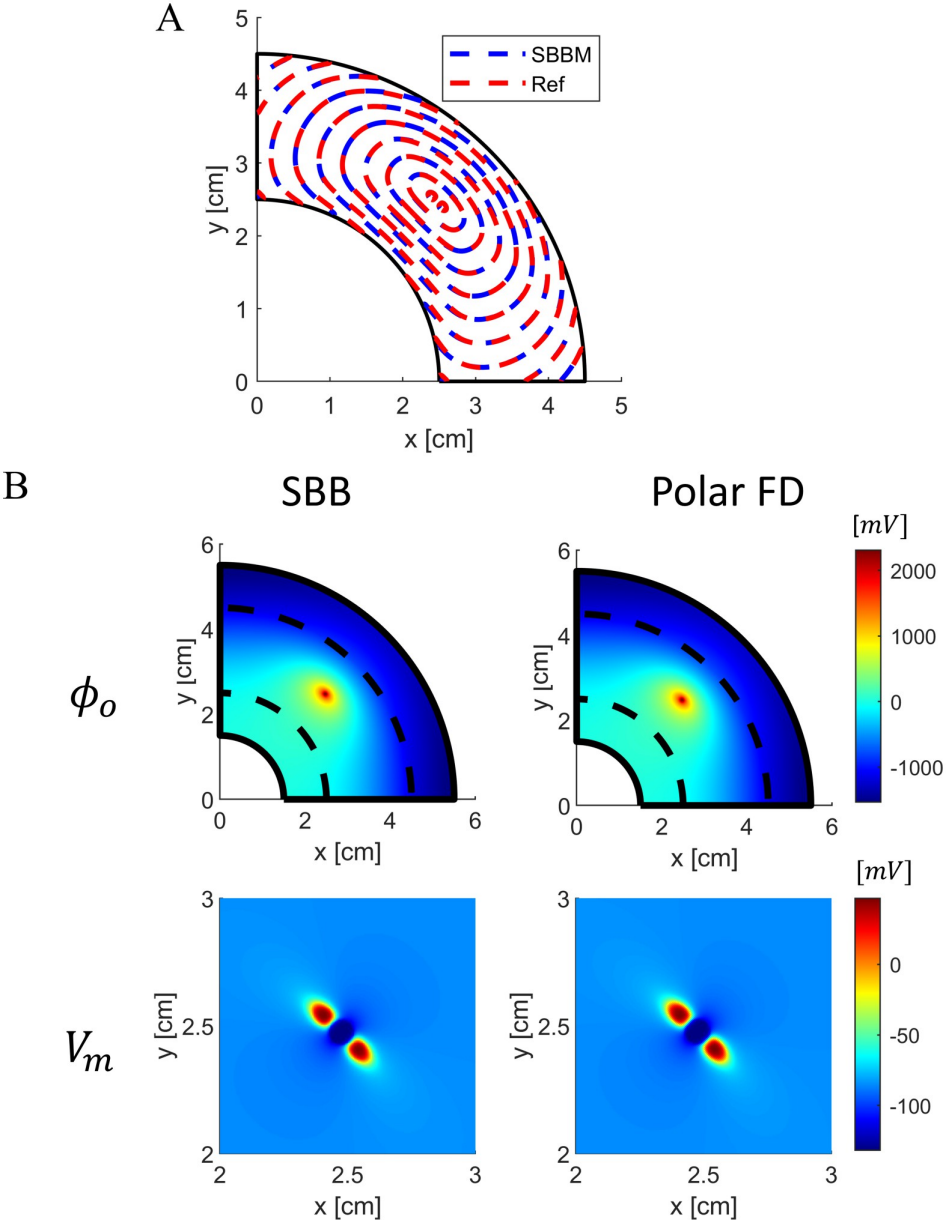
Comparison between SBB and polar FD methods in a 2D anisotropic annular geometry with anodic extracellular stimulation. A) Contour plots of membrane potential (*V*_*m*_ = −40 *mV*) at different times for the SBB and polar FD methods. B) Comparison of extracellular potential field (first line) and membrane potential (second line) obtained with SBB and polar FD methods at two different time instants. Dashed black lines indicate the boundary between the cardiac tissue and the external conductor. Membrane potential is zoomed at the stimulation region.

To assess the accuracy of the SBB method in simulating more complex scenarios, we also stimulated the annular anisotropic tissue with a strong unipolar anodic point source to induce virtual electrode polarization. Again, we observed a good agreement in wavefront velocity (Fig 6A). Additionally, Fig 6B shows the membrane potential and the extracellular potential during the anodic stimulation obtained with the SBB and the polar FD methods. The SBB method demonstrated to accurately reproduce the virtual electrode polarization phenomenon.

**Fig 7:**
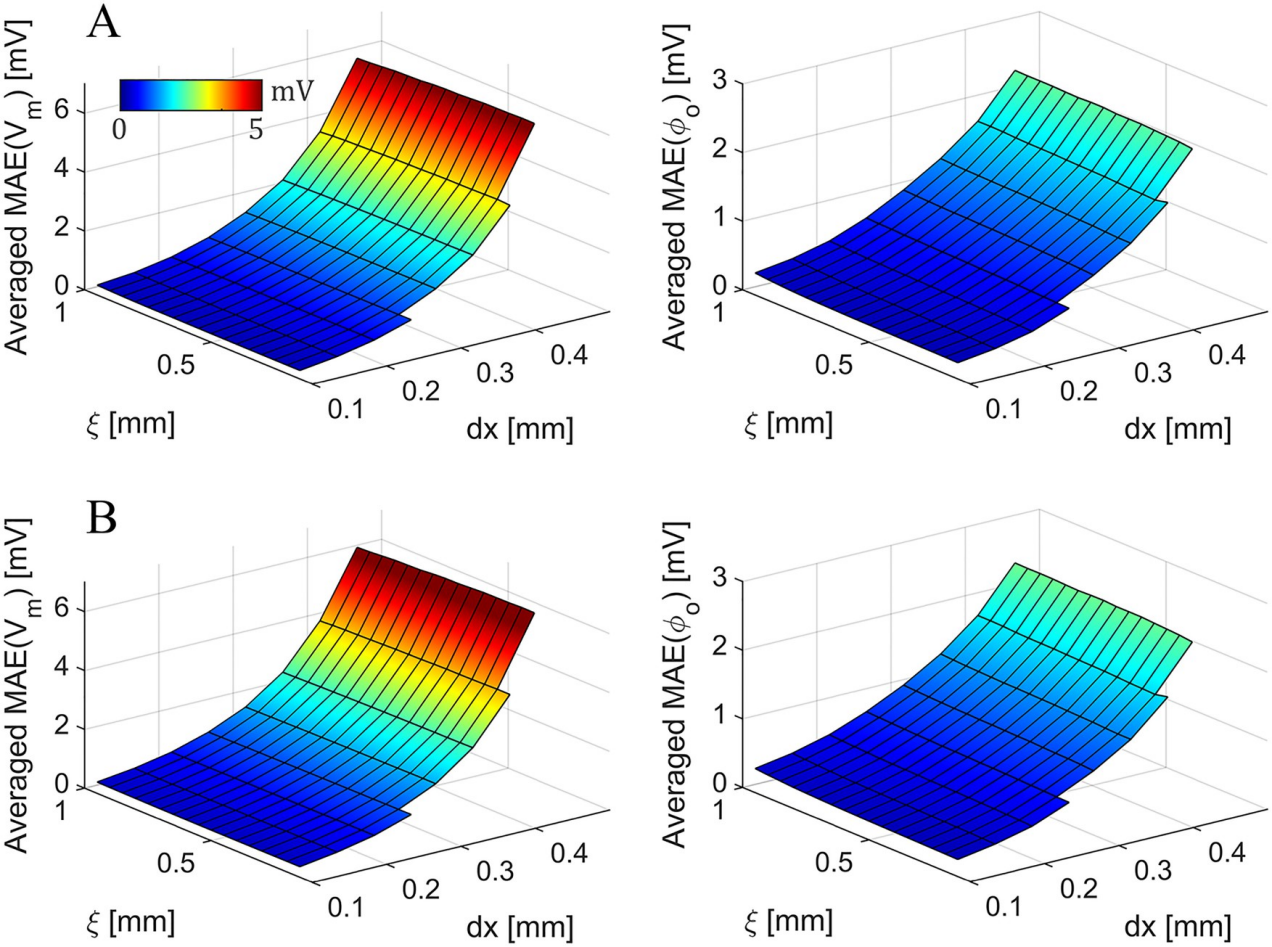
Grid convergence tests of the SBB method for different values of *ξ* with and without operator splitting in the 2D annular geometry. A) Time-averaged mean absolute error in membrane potential and extracellular potential obtained with the SBB method without operator splitting for different values of *ξ* and *dx*. Missing elements indicate that the SBB algorithm without operator splitting did not achieve a solution. B) Time-averaged mean absolute error in membrane potential and extracellular potential obtained with the SBB method exploiting operator splitting for different values of *ξ* and *dx*.

To ensure the convergence of the numerical method, we carried out an additional convergence test in the 2D annular geometry (Fig 7). We repeated the simulation showed in Fig 5 with the SBB method for *dx* varying from 0.01 to 0.045, and *ξ* from 0.015 to 0.1, both in steps of 0.005. For each pair (*dx*, *ξ*), we compared the SBB solution with a reference solution obtained by solving bidomain equations with the standard FD method in polar coordinates (*dr* = 0.1 *mm*, *dθ* = 0.0029). Similarly to the 1D case, the grid convergence test showed that the SBB method converges to the reference solution when *dx* and *ξ* approach to 0 (Fig 7A). Note that the MAE values are lower with respect to the 1D case, probably due to the lower intensity of the stimulation, which is applied in the center of the annular model. Furthermore, we performed the same analysis by using the SBB method exploiting operator splitting (Fig 7B). The results showed that the operator splitting technique does not introduce additional significant source of errors, even if the discrepancy with the reference solution is slightly higher in this case.

### 3D anatomically detailed model

**Fig 8:**
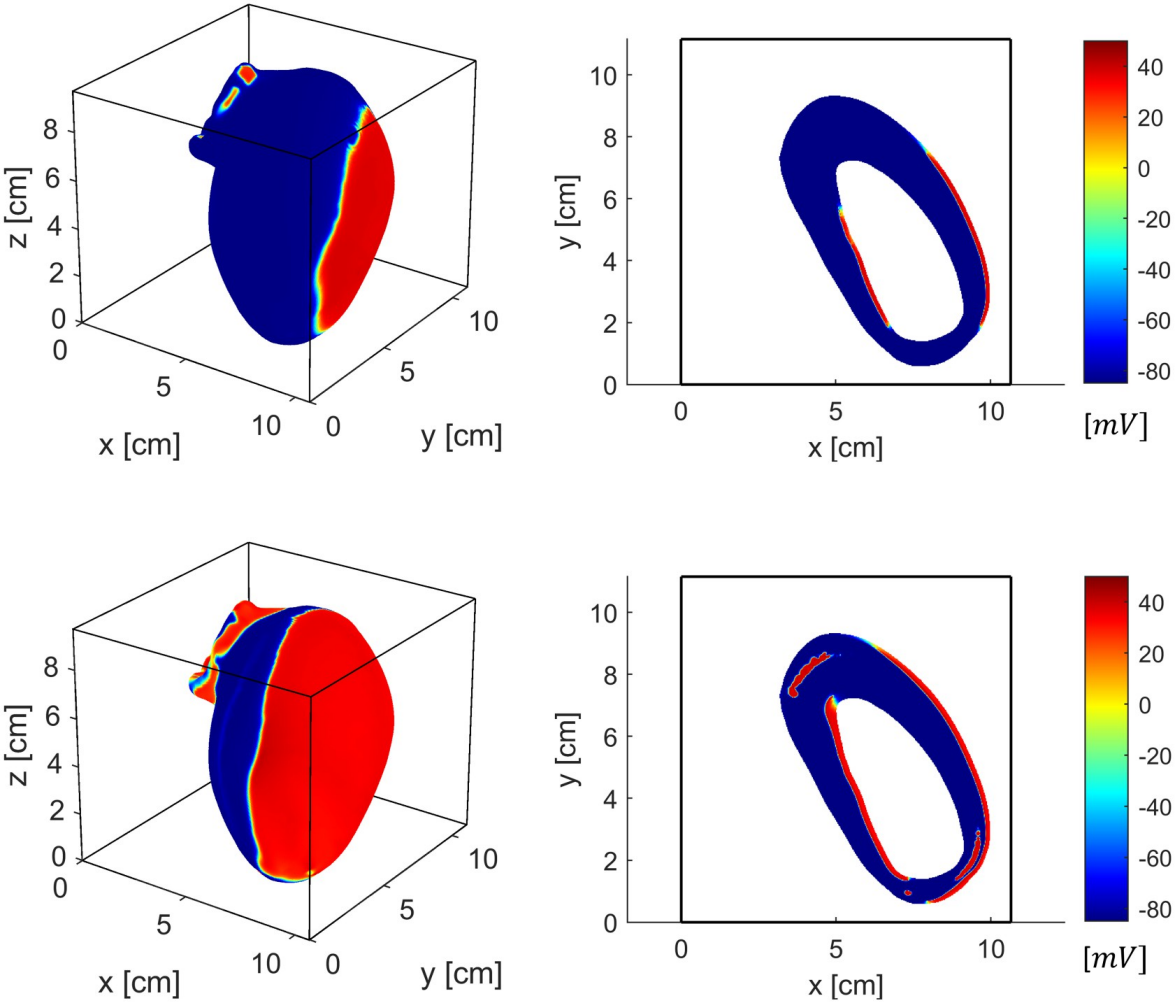
3D anatomically detailed human cardiac simulation with a weak (top) and strong (bottom) defibrillation shock. A slice of the left ventricle (z = 2.25 cm) is shown on the right side. The weak shock induces membrane depolarization at cardiac surfaces, mainly on the epicardial site. Virtual electrodes were generated around the large valve openings. The strong shock induces larger depolarized regions with virtual electrodes located also inside the myocardium.

We tested the SBB method in a human left ventricle geometry with fiber anisotropy. We applied a square waveform defibrillation shock between two electrode plates located at two opposite boundaries of the bath (the anode was placed in the plane x = 0). We applied two shocks of different intensity: a weak shock (i.e., 1.5 A) and a strong shock (i.e., 6 A). Fig 8 shows the membrane potential in the cardiac tissue at 1 ms after the weak (top) or strong (bottom) stimulation. The weak shock induces membrane depolarization at the cardiac surfaces (endocardial and epicardial), which then propagates in the rest of the myocardium. The depolarized regions are larger in the epicardial surface. Additionally, virtual electrodes were generated around aortic and mitral valve openings, and contributed to the depolarization of the cardiac tissue. The strong shock induces larger depolarized areas both in the epicardial and endocardial regions. Additionally, virtual electrodes around valve openings extend over a larger area and are more than those induced by the weak shock. Furthermore, additional virtual electrodes were generated inside the ventricular wall, far from cardiac surfaces. This phenomenon, known as bulk virtual electrode polarization, has been already reported in previous cardiac models based on animal geometries [15, 54, 55]. Indeed, previous works reported that unequal anisotropy ratios is a necessary condition for the generation of bulk virtual electrode polarization in the myocardial wall [15].

## Conclusion

In this manuscript, we describe a novel FD method for cardiac bidomain simulations. The proposed method employs a smoothed boundary representation of the cardiac geometries to accurately implement the bidomain boundary conditions without the need of a structured mesh that explicitly tracks the heart-torso interfaces. Indeed, the main advantage of the SBB method is the accurate implementation of bidomain equations directly on voxel structures acquired with imaging techniques. Additionally, the employment of a cartesian grid facilitates the implementation and parallelization of numerical schemes [56]. We reported some significant examples assessing the method’s accuracy using nontrivial test geometries and demonstrating the applicability of the method to complex anatomically detailed human cardiac geometries. In particular, we showed that the SBB method could be employed to simulate cardiac defibrillation in a human left ventricle. It is worth mentioning that accurate modelling of cardiac defibrillation requires additional details we have not considered in our proof-of-concept simulations, whose scope was only to provide evidence of the feasibility of the SBB method in large anatomically detailed geometries. First, the ionic model we used does not include an electroporation current, which is important in cardiac defibrillation [27]. Second, the vascular structure may play an important role in the defibrillation process, especially for large cardiac vessels [54]. However, accurate implementation of physiological details is beyond the scope of this work. Despite the advantages of the SBB method, a smoothed boundary representation of fine anatomical structures (e.g., small blood vessels) may be challenging due to the use of a regular grid. Nevertheless, the role of fine and isolated anatomical structures is likely to be negligible [54]. Additionally, it would be possible to better represent fine anatomical structures by employing a grid refinement procedure [56]. In this work, we employed a simple FD scheme for spatial discretization, however the SBB approach is independent of the numerical scheme used. Indeed, other choices that better deal with anisotropic diffusion are likely to be more accurate (see e.g., [31]). Similarly, we employed the standard ILU preconditioner, whereas other more efficient solutions exist. In this regard, it is worth mentioning that, by using regular grids, the SBB method can be easily coupled with a geometric multigrid preconditioner, significantly improving computational speed [41]. Alternatively, algebraic multigrid preconditioners (e.g., [51]) could be used to speed up numerical convergence. Thanks to the employment of regular grids, the SBB method could also be combined with adaptive mesh refinement algorithms avoiding the construction of a new unstructured mesh at each iteration [56]. We believe that the SBB method offers a simple solution for solving bidomain equations in anatomical models without the need of unstructured grids. Given the ease of implementation, the SBB method provides an attractive and feasible alternative to finite element methods, and could find application in future research guiding electrotherapy with computational models (e.g., [57, 58]).

## Supporting information

S1 Appendix Proof of the convergence and numerical implementation of the SBB model. The appendix reports a simple proof of the convergence of the SBB model to the bidomain boundary conditions in the 1D case and provides additional details regarding the numerical scheme used in this work.
(PDF)
