# Supplementary material for: A smoothed boundary bidomain model for cardiac simulations in anatomically detailed geometries
Source: PLoS One. 2023 Jun 9;18(6):e0286577. doi: 10.1371/journal.pone.0286577 (PMC10256234; doi:10.1371/journal.pone.0286577)
Supplement: S1 Appendix — The appendix reports a simple proof of the convergence of the SBB model to the bidomain boundary conditions in the 1D case and provides additional details regarding the numerical scheme used in this work. (PDF) [file pone.0286577.s001.pdf]

A smoothed boundary bidomain model for cardiac simulations in anatomically detailed geometries

S1 Appendix

Niccolò Biasi[1*], Paolo Seghetti[2,3], Matteo Mercati[1], Alessandro Tognetti[1,4]

1 Information Engineering Department, University of Pisa, Pisa, Italy
2 Health Science Interdisciplinary Center, Scuola Superiore Sant'Anna, Pisa, Italy
3 National Research Council, Institute of Clinical Physiology, Pisa, Italy
4 Research Centre "E. Piaggio", University of Pisa, Pisa, Italy

* niccolo.biasi@phd.unipi.it

The aim of this document is to prove the convergence of the smoothed boundary bidomain model to the bidomain boundary conditions in the 1D case, and to provide additional details about the numerical scheme used.

Proof of the convergence of the smoothed boundary bidomain model to the bidomain boundary conditions

For the convenience of the reader, we reported here the smoothed boundary bidomain equations (equations 13 and 15 in the manuscript):

$$\psi \frac{\partial V_m}{\partial t} - \nabla \cdot \left(\psi D_i \nabla \left(V_m + \phi_o\right)\right) = -\psi I_{ion} \tag{S1.1}$$

$$\nabla \cdot \left(\psi D_i \nabla V_m\right) + \nabla \cdot \left(\left(\psi \left(D_i + D_o\right) + \left(1 - \psi\right) D_t\right) \nabla \phi_o\right) = I_{ext} \tag{S1.2}$$

Considering the 1D case and integrating equation S1.1 over the interfacial region, we obtain:

$$\int_{a-\xi}^{a+\xi} \psi \left(\frac{\partial V_m}{\partial t} + I_{ion}\right) dx = \int_{a-\xi}^{a+\xi} \frac{\partial}{\partial x} \left(\psi D_i \frac{\partial}{\partial x} \left(V_m + \phi_o\right)\right) dx \tag{S1.3}$$

where a is the left edge of the 1D domain and $a - \xi < x < a + \xi$ is the interfacial region. Since $\psi|_{a-\xi} = 0$ and $\psi|_{a+\xi} = 1$, we can write:

$$\int_{a-\xi}^{a+\xi} \psi \left(\frac{\partial V_m}{\partial t} + I_{ion}\right) dx = D_i \frac{\partial}{\partial x} \left(V_m + \phi_o\right)\bigg|_{a+\xi} \tag{S1.4}$$

Considering the limit $\xi \to 0$ and the mean value theorem of integrals, we can neglect the spatial variation of the left-hand side:

$$D_i \frac{\partial}{\partial x} \left(V_m + \phi_o\right)\bigg|_{a} = h_0 \xi \tag{S1.5}$$

where h_0 is a constant. Thus, in the sharp interface limit $\xi \to 0$, the boundary condition 8 in the manuscript is recovered.

Similarly, integrating equation S1.2 over the interfacial region, we obtain:

$$D_i \frac{\partial V_m}{\partial x}\bigg|_{a+\xi} + (D_i + D_o)\frac{\partial \phi_o}{\partial x}\bigg|_{a+\xi} - D_t \frac{\partial \phi_o}{\partial x}\bigg|_{a-\xi} = h_1 \xi. \tag{S1.6}$$

where h_1 is a constant. Thus, in the sharp interface limit $\xi \to 0$, the sum of the boundary conditions 6 and 8 in the manuscript is recovered. Therefore, we proved that in the 1D case, in the limit $\xi \to 0$, the smoothed boundary bidomain equations S1.2 and S1.1 implicitly implement bidomain boundary conditions 6 and 8.

Numerical scheme

The matrix $\boldsymbol{A_s}$ in equation 18 of the manuscript implements the operator $\nabla \cdot (\psi D_i \nabla)$ through a standard finite difference scheme. The diffusion term $S_1 = \nabla \cdot (\psi D_i V_m)$ can be written as a sum of doubly repeated indices, where we introduced the terms j and k to represent two of either x, y or z:

$$S_1 = \sum_j \sum_k \frac{\partial}{\partial x_j}\left(\psi d^i_{jk} \frac{\partial V_m}{\partial x_k}\right) \tag{S1.7}$$

where d^i_{jk} are the elements of the diffusion tensor D_i. The derivatives in the equations can be expanded as follows:

$$S_1 = \sum_j \sum_k \frac{\partial \psi}{\partial x_j} d^i_{jk} \frac{\partial V_m}{\partial x_k} + \psi \sum_j \sum_k d^i_{jk} \frac{\partial^2 V_m}{\partial x_j \partial x_k} + \psi \sum_j \sum_k \frac{\partial d_{jk}}{\partial x_j}\frac{\partial V_m}{\partial x_k} \tag{S1.8}$$

All the derivatives in the above equation were discretized for each element with the central finite difference approximation. The coefficients multiplying the elements of $\boldsymbol{V_m}$ are the elements of the matrix $\boldsymbol{A_s}$. Notably, the derivatives of ψ and d^i_{jk} can be precomputed and saved without the need to be recomputed at each time step. Similarly, the matrix $\boldsymbol{B_s}$ in equation 19 implements the operator $\nabla \cdot ((\psi (D_i + D_o) + (1-\psi) D_t) \nabla)$. The associated diffusion term $S_2 = \nabla \cdot ((\psi (D_i + D_o) + (1-\psi) D_t) \nabla \phi_o)$ can be rewritten as:

$$S_2 = \sum_j \sum_k \frac{\partial}{\partial x_j}\left(\left(\psi \left(d^i_{jk} + d^o_{jk}\right) + (1-\psi)\, d^t_{jk}\right) \frac{\partial \phi_o}{\partial x_k}\right) \tag{S1.9}$$

where d^o_{jk} and d^t_{jk} are the elements of the diffusion tensors D_o and D_t, respectively. The derivatives in the equations can be expanded as follows:

$$\begin{aligned} S_2 = &\sum_j \sum_k \frac{\partial \psi}{\partial x_j}\left(d^i_{jk} + d^o_{jk}\right)\frac{\partial \phi_o}{\partial x_k} + \psi \sum_j \sum_k \left(\frac{\partial d^i_{jk}}{\partial x_j} + \frac{\partial d^o_{jk}}{\partial x_j}\right)\frac{\partial \phi_o}{\partial x_k} \\ &+ \psi \sum_j \sum_k \left(d^i_{jk} + d^o_{jk}\right)\frac{\partial^2 \phi_o}{\partial x_j \partial x_k} - \sum_j \sum_k \frac{\partial \psi}{\partial x_j} d^t_{jk} \frac{\partial \phi_o}{\partial x_k} \\ &+ (1-\psi)\sum_j \sum_k \frac{\partial d^t_{jk}}{\partial x_j}\frac{\partial \phi_o}{\partial x_k} + (1-\psi)\sum_j \sum_k d^t_{jk} \frac{\partial^2 \phi_o}{\partial x_j \partial x_k} \end{aligned} \tag{S1.10}$$

Again, all the derivatives were approximated with central finite differences, and the coefficients multiplying the elements of $\boldsymbol{\phi_o}$ are the elements of $\boldsymbol{B_s}$.
